# Supplementary material for: Antibiotic resistance plasmids in Enterobacteriaceae isolated from fresh produce in northern Germany
Source: Microbiol Spectr. 2024 Sep 17;12(11):e00361-24. doi: 10.1128/spectrum.00361-24 (PMC11537058; doi:10.1128/spectrum.00361-24)
Supplement: Table S1 — Complete genome sequences of Enterobacteriaceae. [file spectrum.00361-24-s0001.docx]

**Supplementary Table S****1:** Complete genome sequences of *Enterobacteriaceae* were deposited in the GenBank/ENA/DDBJ databases.

| **Strain** | **Accession-number** | **Plasmid** | **Accession-number** |
| --- | --- | --- | --- |
| Cigi1 | CP115035 | pCIGI1_1 | CP115036 |
|  |  | pCIGI1_2 | CP115037 |
|  |  | pCIGI1_3 | CP115038 |
| Cipo4 | CP126611 | pCIPO4_1 | CP126612 |
|  |  | pCIPO4_2 | CP126613 |
| Ciw5.1 | CP126605 | pCIW5.1_1 | CP126606 |
|  |  | pCIW5.1_2 | CP126607 |
| Ciw5.2 | CP115032 | pCIW5.2_1 | CP115033 |
|  |  | pCIW5.2_2 | CP115034 |
| Cipa6.1 | CP115031 |  |  |
| Cipa6.2 | CP115030 |  |  |
| Cif11 | CP126623 | pCIF11_1 | CP126624 |
| Cipo13 | CP115024 | pCIPO13_1 | CP115025 |
|  |  | pCIPO13_2 | CP115026 |
|  |  | pCIPO13_3 | CP115027 |
|  |  | pCIPO13_4 | CP115028 |
|  |  | pCIPO13_5 | CP115029 |
| Endy1 | CP126604 |  |  |
| Endy2 | CP126610 |  |  |
| Enh11 | CP126608 | pENH11_1 | CP126609 |
| Enb12 | CP115601 | pENB12_1 | CP115602 |
| Ec1115 | CP126614 | pEC1115_1 | CP126615 |
|  |  | pEC1115_2 | CP126616 |
|  |  | pEC1115_3 | CP126617 |
|  |  | pEC1115_4 | CP126618 |
| Ec1117 | CP126602 | pEC1117_1 | CP126603 |
| Ec1119 | CP126600 | pEC1119_1 | CP126601 |
| Ec1120 | CP126598 | pEC1120_1 | CP126599 |
| Kgr1 | CP126593 | pKGR1_1 | CP126594 |
|  |  | pKGR1_2 | CP126595 |
|  |  | pKGR1_3 | CP126596 |
|  |  | pKGR1_4 | CP126597 |
| Kva3 | CP126619 |  |  |
| Kpneu4 | CP115021 | pKPNEU4_1 | CP115022 |
|  |  | pKPNEU4_2 | CP115023 |
| Kpneu8 | CP126622 | pKPNEU8_1 | CP126620 |
|  |  | pKPNEU8_2 | CP126621 |
| Kpneu28 | CP136465 | pKPNEU28_1 | CP136461 |
|  |  | pKPNEU28_2 | CP136462 |
|  |  | pKPNEU28_3 | CP136463 |
|  |  | pKPNEU28_4 | CP136464 |
| Kpneu34 | CP136460 | pKPNEU34_1 | CP136456 |
|  |  | pKPNEU34_2 | CP136457 |
|  |  | pKPNEU34_3 | CP136458 |
|  |  | pKPNEU34_4 | CP136459 |
